# Emerging sports footwear technologies and their effects on running economy, biomechanics, and performance: a systematic review

**DOI:** 10.1186/s13102-026-01721-w

**Published:** 2026-05-04

**Authors:** Cristina Ioan Alexe, Prashant Kumar Choudhary, Suchishrava Choudhary, Sohom Saha, Elena Adelina Panaet, Dan Iulian Alexe

**Affiliations:** 1 Department of Physical Education and Sports Performance, “Vasile Alecsandri” University of Bacau, Bacau, 600115 Romania; 2 Department of Physical Education Pedagogy, Lakshmibai National Institute of Physical Education, Gwalior, Madhya Pradesh 474002 India; 3 Department of Sports Psychology, Lakshmibai National Institute of Physical Education, Gwalior, Madhya Pradesh 474002 India; 4 Department of Physical and Occupational Therapy, “Vasile Alecsandri” University of Bacau, Bacau, 600115 Romania

**Keywords:** Running economy, Carbon-fibre plate shoes, Sports footwear technology, Running biomechanics, Endurance performance, Midsole foams

## Abstract

Advances in running footwear technology, particularly carbon-fibre plates and highly resilient midsole foams, have been proposed to enhance running economy and performance. However, evidence remains heterogeneous and context-dependent.

**Purpose**

This systematic review synthesised empirical evidence on the biomechanical, physiological, and performance effects of emerging footwear technologies. A PRISMA-guided search identified 14 experimental studies examining footwear effects on running economy, biomechanics, and physiological responses. Methodological quality and risk of bias were assessed using a modified approach based on the Cochrane Risk of Bias framework, adapted to accommodate the diversity of study designs included in this review, including randomized, non-randomized, and biomechanical simulation studies. Fourteen studies were included. Carbon-fibre plate shoes with resilient midsole foams improved running economy by 2.6–4.2%, with some models reducing metabolic cost by ~ 4% and improving running economy during prolonged running by 2–6%, while increasing lactate-threshold speed by 0.5–0.6 km·h⁻¹ and reducing heart rate (~ 4–5%) and blood lactate (~ 0.3–0.5 mmol·L⁻¹). Biomechanical adaptations included reduced joint work, altered stride mechanics, and improved energy return, whereas maximalist cushioning increased impact loading by 10.7% and loading rate by 12.3%, and carbon-fibre insoles showed no significant performance or metabolic benefits. Modern running footwear technologies can enhance running economy and endurance performance through complex interactions between cushioning properties, plate stiffness, and shoe geometry. Nevertheless, the biomechanical and physiological effects of these technologies are context-dependent. Future research should investigate long-term adaptations, injury risk implications, and individualized footwear design to optimize performance while minimizing injury risk.

## Introduction

Running is one of the most widely practiced physical activities and competitive sports worldwide, attracting participants ranging from recreational joggers to elite endurance athletes. Performance in distance running is strongly influenced by physiological, biomechanical, and technological factors that interact to determine the efficiency of human locomotion. Among these determinants, running economy, defined as the oxygen or metabolic cost required to maintain a given submaximal running velocity, is considered one of the most important predictors of endurance running performance [1, 2]. Improvements in running economy can significantly enhance performance outcomes, particularly in long-distance events where small reductions in metabolic demand can translate into substantial competitive advantages [2].

In recent years, technological innovations in running footwear have emerged as a major factor influencing running biomechanics and physiological efficiency. Advances in materials science, biomechanics, and footwear engineering have led to the development of modern running shoes incorporating features such as carbon-fibre plates, highly resilient midsole foams, increased midsole stiffness, optimized plate geometry, and advanced cushioning systems [3, 4]. These innovations aim to improve running performance by enhancing energy storage and return, modifying joint mechanics, and reducing muscular work during the stance phase of running [5, 6] As a result, footwear technology has become an increasingly important area of investigation within sports biomechanics and sports engineering research.

One of the most influential developments in modern running footwear is the integration of carbon-fibre plates embedded within highly compliant and resilient midsole foams, commonly referred to as “super shoes.” Experimental research has demonstrated that such footwear can substantially reduce the energetic cost of running. For example, Hoogkamer et al. (2018) reported that marathon racing shoes incorporating a curved carbon-fibre plate and a highly resilient PEBA-based foam midsole reduced the metabolic cost of running by approximately 4% compared with conventional racing shoes [7]. Similarly, Barnes and Kilding (2019) observed improvements in running economy ranging from 2.6% to 4.2% when highly trained runners wore advanced footwear technology compared with traditional track spikes or marathon shoes [8]. These improvements are likely mediated by a combination of biomechanical and energetic mechanisms, including increased longitudinal bending stiffness, improved energy return from the midsole foams, and altered lower-limb joint mechanics [9].

Beyond metabolic improvements, footwear technology has also been shown to influence running biomechanics and muscle-tendon mechanics. Studies examining increased midsole bending stiffness have reported reductions in arch deformation and modifications in muscle-tendon unit behavior, which may improve locomotor efficiency by reducing muscular work during ground contact [10]. Similarly, research investigating variations in carbon-fibre plate geometry suggests that curved plates may reduce joint moments at the hip and knee while improving load distribution across the forefoot [11, 12]. Such biomechanical adaptations may not only enhance running performance but may also influence injury risk by altering the distribution of mechanical loads across the lower extremities [13].

Despite these promising findings, the influence of footwear technology on running performance is complex and not always consistent. Several studies have suggested that the benefits of advanced footwear cannot be attributed to a single design element but instead arise from the combined interaction of multiple structural components, including foam properties, plate stiffness, shoe geometry, and footwear mass [3, 5]. For instance, experimental manipulation of plate stiffness in the Nike Vaporfly shoe demonstrated that reducing longitudinal bending stiffness did not significantly alter running economy, suggesting that performance improvements result from the integrated interaction of plate geometry and midsole foams properties rather than stiffness alone [14]. This concept can be understood as an “integrated footwear system,” in which multiple structural components such as midsole foam properties, plate geometry, stiffness characteristics, and shoe mass interact synergistically to influence biomechanical and physiological responses. Rather than acting independently, these elements collectively determine the overall performance effect of the footwear.

Other footwear designs have produced mixed or context-dependent outcomes. Minimalist footwear, characterized by reduced cushioning and a zero heel-to-toe drop, has been shown to alter running biomechanics by encouraging forefoot strike patterns and reducing impact loading rates [15]. Similarly, studies examining cushioning effects have demonstrated that moderate cushioning may reduce metabolic demand, but excessive cushioning or increased shoe mass may negate these benefits by increasing the metabolic cost of leg swing [16, 17].

Recent research has also highlighted unexpected biomechanical responses to highly cushioned footwear. Kulmala et al. (2018) reported that maximalist running shoes with thick midsoles increased leg stiffness and amplified impact loading during running, suggesting that runners may subconsciously modify their neuromuscular control strategies when running in highly cushioned shoes [18]. These compensatory adaptations may counteract the intended shock-absorbing effects of the footwear and potentially increase the risk of impact-related injuries. Furthermore, the effectiveness of advanced footwear technologies appears to vary depending on running context and environmental conditions. For example, while carbon-plated shoes have demonstrated consistent benefits for road running performance, their effectiveness may be reduced in trail or mountain running environments where uneven terrain alters running mechanics [19]. The findings provide strong evidence that footwear technology interacts with factors such as terrain, running speed, and individual biomechanics to influence performance outcomes. Although the rapid development of innovative running footwear has generated substantial interest among researchers, athletes, and footwear manufacturers, the existing literature remains fragmented across different experimental approaches, biomechanical variables, and footwear technologies. Previous studies have primarily focused on specific shoe components or isolated biomechanical mechanisms, making it difficult to develop a comprehensive understanding of how modern footwear technologies collectively influence running performance and biomechanics. Moreover, variations in study design, participant characteristics, and outcome measures have resulted in inconsistent findings across the literature.

Despite the growing body of research examining the effects of advanced footwear technologies on running performance, several important gaps remain in the literature. First, there is a methodological gap, as existing studies are highly heterogeneous in design, including randomized trials, laboratory experiments, and biomechanical simulations, which limits the ability to draw consistent and generalizable conclusions. Second, there is a biomechanical and physiological inconsistency across studies, with conflicting findings regarding the relative contributions of midsole foams properties, carbon-fibre plate stiffness, and shoe geometry to performance enhancement. Third, there is a contextual gap, as emerging footwear technologies continue to evolve rapidly, yet their effects across different running environments, fatigue conditions, and athlete populations remain insufficiently understood. Therefore, a comprehensive synthesis integrating biomechanical, physiological, and performance outcomes is required to clarify the mechanisms through which modern footwear technologies influence running efficiency and endurance performance.

In response to these gaps, the purpose of the present systematic review was to synthesize existing empirical evidence regarding the biomechanical, physiological, and performance effects of emerging sports footwear technologies in running. Specifically, this study aimed to evaluate how different footwear innovations including carbon-fibre plate designs, advanced midsole foams, midsole stiffness modifications, cushioning systems, and minimalist footwear affect running economy, biomechanical parameters, and endurance performance. By integrating findings from experimental and biomechanical studies, this review seeks to identify key mechanisms through which footwear technologies influence running efficiency and to clarify the conditions under which these technologies provide meaningful performance benefits.

## Materials and methods

### Study selection procedures

**Fig. 1:**
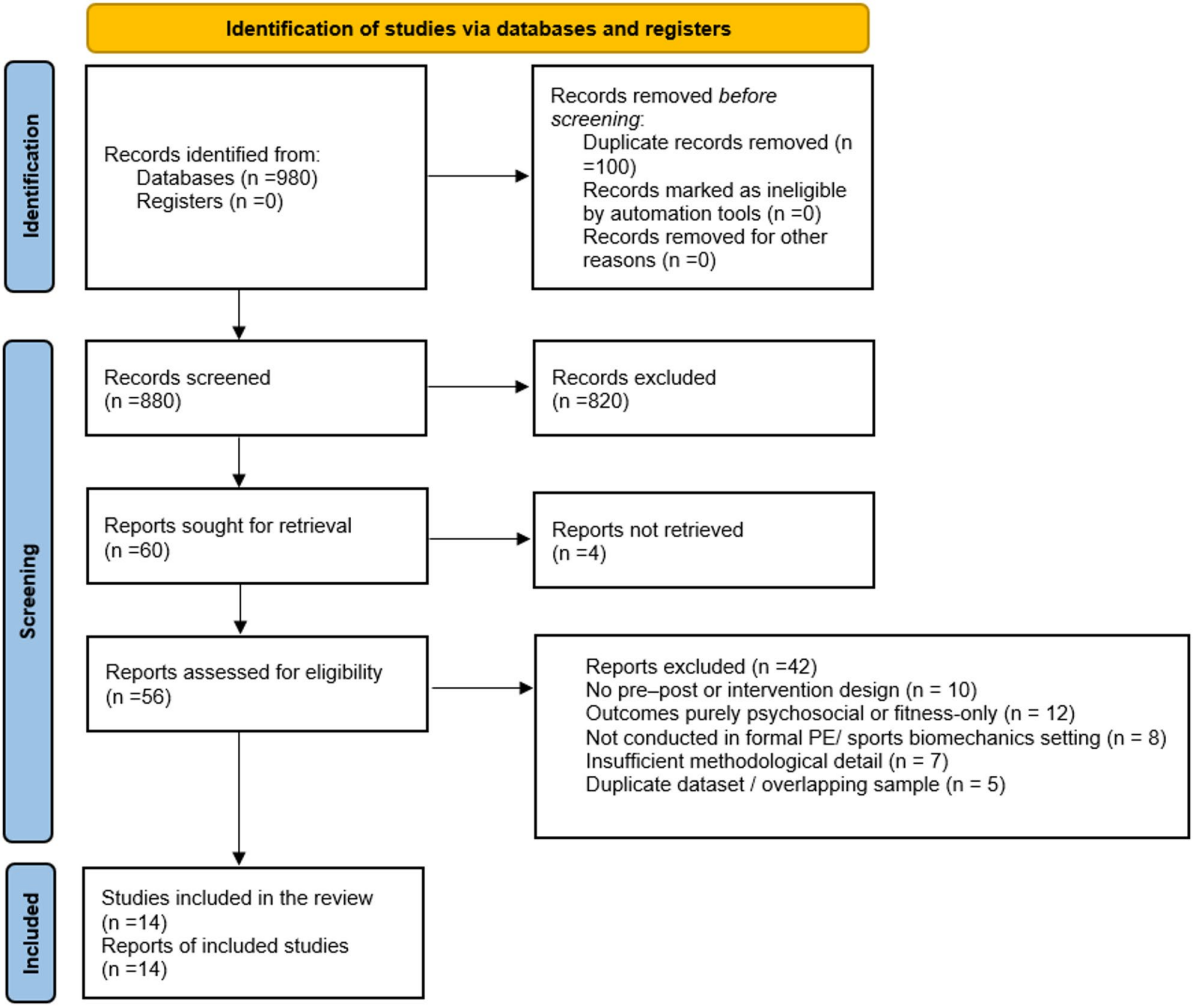
PRISMA 2020 flow diagram illustrating the study selection process for the systematic review

The study selection process followed the methodological framework recommended by the Preferred Reporting Items for Systematic Reviews and Meta-Analyses (PRISMA) guidelines to ensure transparency, reproducibility, and methodological rigour [20, 21]. This systematic review was conducted in accordance with the PRISMA 2020 guidelines. The screening and selection process was conducted independently by two reviewers with expertise in sports biomechanics and systematic review methodology. All identified records were initially screened based on titles and abstracts, followed by full-text evaluation of potentially eligible studies. Disagreements between reviewers at any stage of the selection process were resolved through discussion and consensus. In cases where consensus could not be reached, a third reviewer was consulted to make the final decision. The review aimed to identify empirical studies investigating the effects of emerging sports footwear technologies on running performance, biomechanics, and physiological outcomes. Initially, all potentially relevant records identified through database searches were exported into a reference management system, where duplicate entries were removed. The remaining studies were screened through a two-stage process. In the first stage, titles and abstracts were examined to determine their relevance to the research topic. Studies that clearly did not meet the predefined inclusion criteria were excluded at this stage. In the second stage, the full texts of the remaining articles were assessed in detail against the eligibility criteria defined using the PICO framework (Population, Intervention, Comparison, Outcomes), which is widely used for structuring research questions and eligibility criteria in systematic reviews [22]. Only studies that met al.l inclusion criteria were retained for the final qualitative synthesis. The eligibility criteria required studies to involve human participants engaged in running-related activities and to examine footwear technologies designed to influence running performance or biomechanics. Studies were excluded if they were review articles, conference abstracts without full data, non-peer-reviewed publications, or investigations unrelated to running biomechanics or performance. Studies were included if they explicitly evaluated biomechanical, physiological, or performance outcomes associated with sports footwear interventions in human participants, using experimental, comparative, or biomechanical analysis designs. Studies were excluded if they did not involve footwear-related interventions, lacked objective outcome measures, or were not conducted within a running-specific context. Following this screening process, 14 studies were deemed eligible and included in the final analysis (See Fig. 1).

### Literature search: administration and update

A comprehensive literature search was conducted to identify relevant peer-reviewed studies examining the biomechanical and physiological effects of running footwear technologies. Electronic searches were performed across major scientific databases commonly used in sports science and biomechanics research, including PubMed, Scopus, Web of Science, and Google Scholar. The search strategy employed combinations of keywords and Boolean operators related to footwear technology and running performance, including “running shoes,” “sports footwear technology,” “carbon-fibre plate running shoes,” “running economy,” “running biomechanics,” “midsole stiffness,” “minimalist footwear,” “cushioning,” and “running performance.” The search was restricted to peer-reviewed articles published in English. In addition to database searches, the reference lists of relevant studies were manually screened to identify additional potentially eligible articles that may not have been captured during the initial search. The literature search was conducted and updated before the final data synthesis to ensure the inclusion of the most recent studies, and all retrieved records were evaluated according to the predefined inclusion and exclusion criteria (See Table 1). A detailed Boolean search strategy was developed to ensure comprehensive retrieval of relevant studies. An example of the search string used in PubMed is as follows: (“running shoes” OR “sports footwear” OR “footwear technology” OR “carbon fibre plate” OR “carbon-fibre plate” OR “advanced footwear”) AND (“running economy” OR “biomechanics” OR “gait” OR “ground reaction force” OR “metabolic cost” OR “performance”) AND (“running” OR “endurance running” OR “distance running”). Where applicable, Medical Subject Headings (MeSH) terms and database-specific indexing terms were incorporated to enhance search sensitivity. Filters were applied to include studies published in English, involving human participants, and available as full-text peer-reviewed articles. The search was limited to studies published between January 2000 and March 2026 to capture developments in modern footwear technologies. No additional restrictions were applied beyond the predefined publication period, ensuring a comprehensive inclusion of studies relevant to modern footwear technologies.

**Table 1:** Inclusion and exclusion criteria

PICO Component	Inclusion Criteria	Exclusion Criteria
Population (P)	Human participants involved in running-related activities, including recreational runners, trained runners, competitive runners, or elite runners. Studies including both male and female adults (≥ 18 years) with experience in treadmill or overground running.	Studies involving non-human subjects, children or adolescents (< 18 years), clinical populations unrelated to running performance (e.g., neurological disorders), or participants not engaged in running activities (e.g., walking-only studies).
Intervention (I)	Studies investigating sports footwear technologies specifically designed to modify running performance or biomechanics. Eligible technologies include carbon-fibre plates, advanced midsole foams (e.g., PEBA/ZoomX), minimalist footwear, maximalist cushioning systems, innovative midsole structures, altered heel-toe drop, and footwear stiffness modifications.	Studies that do not investigate footwear technology (e.g., orthotics unrelated to sports shoes, training programs, or gait retraining without footwear manipulation). Studies evaluating non-running footwear, such as casual shoes, occupational boots, or clinical orthopaedic devices.
Comparison (C)	Studies comparing two or more footwear conditions, including comparisons between advanced footwear technology and conventional running shoes, minimalist vs. cushioned footwear, barefoot vs. shod running, or different shoe structural designs (e.g., curved vs. flat carbon plates).	Studies lacking a control or comparison condition, including single-condition observational studies, descriptive reports, or case studies without footwear comparison.
Outcomes (O)	Studies reporting quantitative biomechanical, physiological, or performance outcomes related to running. These include: running economy (VO₂ or metabolic cost), ground reaction forces, loading rate, joint kinematics and kinetics, plantar pressure, muscle activation (EMG), stride parameters (step length, cadence, contact time), physiological responses (heart rate, lactate), or running performance measures (time trial performance).	Studies not reporting objective biomechanical or physiological outcomes, such as purely subjective surveys, footwear preference questionnaires, or marketing-based evaluations without measurable performance or biomechanical variables.
Study Design	Experimental, quasi-experimental, randomized crossover, randomized controlled trials, or biomechanical laboratory experiments involving footwear testing during running tasks.	Review articles, systematic reviews, meta-analyses, bibliometric analyses, conference abstracts without full data, editorials, commentaries, and narrative reviews.
Publication Characteristics	Peer-reviewed articles published in scientific journals in English.	Grey literature, unpublished theses, non-peer-reviewed reports, and articles not available in English.
Sport Context	Studies conducted in running contexts, including treadmill running, overground running, road running, or trail running simulations.	Studies conducted in other sports contexts (e.g., basketball, soccer, walking biomechanics) unless running biomechanics was specifically evaluated.

Although the primary inclusion criteria focused on studies directly examining footwear interventions with comparative designs, certain studies employing hybrid or complementary approaches were also included to provide a comprehensive understanding of biomechanical mechanisms. For example, studies combining gait retraining with minimalist footwear and biomechanical simulation models were retained where they provided relevant mechanistic insights into footwear-related adaptations. Accordingly, the eligibility criteria were interpreted with a degree of flexibility to incorporate emerging and interdisciplinary research designs relevant to the review objectives.

### Data extraction

Data extraction was conducted using a standardized extraction framework to ensure consistency and accuracy across the included studies. For each eligible study, relevant information was systematically collected and organised into structured tables. The extracted data included study identification (authors and year of publication), country of study, participant characteristics (such as sample size, sex, and training status), study design, footwear technology investigated, key outcome measures, and the main findings related to biomechanics, physiological responses, or running performance. These extracted data formed the basis for Table 2 (Characteristics of Included Studies) and Table 4 (Outcome Measures and Direction of Effects Across Included Studies). Where necessary, additional details regarding biomechanical variables, metabolic indicators, and performance outcomes were also extracted to support the synthesis and interpretation of results.

**Table 2:** Characteristics of included studies *n* = 14

No.	Study (Author, Year)	Country	Participants	Study Design	Footwear Technology Investigated	Key Outcome Measures	Main Findings
1	Hoogkamer et al., (2018) [7]	USA	18 male high-caliber runners (average *V*˙*O*2*max*​: 72.1 mL *O*2​/kg/min)	Within-subject repeated measures design comparing three shoe models across three running velocities (14, 16, and 18 km/h)	Nike prototype shoes (NP) featuring a highly compliant and resilient PEBA (ZoomX) foam midsole combined with a full-length, curved carbon-fibre plate	Energetic cost of running (W/kg), biomechanical metrics (peak vertical force, step frequency, contact time), and shoe mechanical properties (compliance and resilience)	The prototype shoes reduced the energetic cost of running by 4% on average compared to established racing shoes (Nike Zoom Streak 6 and adidas adizero Adios BOOST 2),. These savings were observed in all participants and were independent of running velocity
2	Barnes & Kilding, (2019) [8]	USA & New Zealand	24 highly-trained runners (12 male, 12 female)	Randomized crossover design	Nike Vaporfly (NVF), Nike Zoom Matumbo 3 (NZM track spikes), Adidas Adizero Adios 3 (ADI marathon shoes), and weight-matched NVF (NVF+).	Running economy (VO2, carbon dioxide production) and biomechanical measures.	NVF improved running economy by 2.6% vs. track spikes, 4.2% vs. marathon shoes, and 2.9% vs. weight-matched shoes. It is considered a viable option for track and road racing
3	Healey & Hoogkamer, (2022) [14]	USA	17 healthy male runners (average age 24, weight 67.8 kg)	Metabolic and biomechanical protocols using a randomized, mirrored order with blinded participants	Longitudinal bending stiffness (LBS) of the curved carbon-fibre plate in Nike Vaporfly 4% shoes (Intact vs. Cut)	Running economy (metabolic rate), ground reaction forces (GRF), and lower limb joint mechanics (knee, ankle, and MTP joints)	Reducing LBS did not significantly affect running economy (0.55% difference). Biomechanical differences were mainly in the MTP joint, with increased dorsiflexion and negative work in the cut shoes. Energy savings likely result from a combination of foam, geometry, and the plate rather than LBS alone
4	Corbí-Santamaría, P., et al. (2025) [30]	Spain	12 well-trained male mountain runners (average age: 35.4 years; experience: 7.9 years)	A 3-day randomized crossover experiment involving a maximal aerobic speed test, a circuit familiarization session, and two 5.19 km mountain circuit time trials performed in a randomized order with 30 min of passive recovery	Advanced Footwear Technology (AFT) featuring rigid carbon fiber plates and reactive midsole foams compared against conventional (CON) mountain running shoes	Performance (time, power), physiological variables (heart rate, blood lactate), running biomechanics (step frequency, step length, contact time, vertical oscillation of the center of gravity, leg and vertical stiffness), and perceived comfort	The use of AFT did not enhance performance or physiological responses during the simulated mountain race, regardless of whether the terrain was uphill, downhill, or mixed. However, AFT significantly altered running biomechanics by reducing step frequency and increasing vertical oscillation of the center of gravity. While overall comfort remained similar, AFT shoes were perceived as significantly less flexible than conventional shoes .
5	Yang et al. (2020) [31]	China	17 male recreational runners completed the intervention (9 in the gait retraining group, 8 in the minimalist shoe only group)	Parallel randomized control design with a 12-week intervention.	Minimalist footwear (INOV-8 Bare-XF 210 V2) with a 3 mm outsole, no midsole, and 0 mm heel-toe drop	Impact forces (loading rate, peak impact force), joint mechanics (kinematics/kinetics of the hip, knee, and ankle), and vertical stiffness	The 12-week program converted 78% of rearfoot strikers into forefoot strikers in the gait retraining group. This combination more effectively reduced the loading rate and avoided peak impact forces than minimalist shoes alone. Both groups showed improved vertical stiffness, which may enhance running economy
6	Xu et al. (2025) [32]	China	12 male elite runners (aged 21.8 ± 1.3 years; personal best half marathon sub-85 min or full marathon sub-180 min)	Randomized crossover design using two-way repeated measures ANOVA and Statistical Parametric Mapping (SPM) to compare pre- and post-fatigue biomechanics	Carbon-fibre plate (CFP) shapes: Flat CFP shoes (ASICS METASPEED SKY PARIS) vs. Curved CFP shoes (ASICS METASPEED EDGE PARIS)	Lower limb kinematics (joint angles), kinetics (joint moments, power, work, VALR), and muscle activation (EMG of tibialis anterior, medial/lateral gastrocnemius)	Curved CFP shoes significantly reduced hip and knee contact angles and hip flexion moments compared to flat CFP shoes. An interaction effect showed tibialis anterior (TA) activation was higher in curved shoes pre-fatigue but lower post-fatigue compared to flat shoes. The curved plate altered the forefoot bending angle, potentially enhancing performance and reducing injury risk
7	Cigoja et al. (2020) [10]	Canada	13 male, recreational runners	Randomized Crossover Trials	Increased Midsole Bending Stiffness (MBS) achieved by inserting carbon fibre plates into a control shoe	Foot arch deformation (angle and velocity); plantar muscle-tendon unit (pMTU) and shank muscle-tendon unit (sMTU) shortening and shortening velocity; positive, negative, and network at these structures	Increased MBS resulted in significantly less arch deformation and reduced pMTU shortening. Shortening velocities for both the pMTU and sMTU were lower compared to the control shoe,. Additionally, positive and net work at the arch and pMTU, as well as net work at the sMTU, were significantly reduced.
8	Fu et al. (2022) [33]	China	15 healthy female runners (all identified as rearfoot strikers)	Experimental biomechanical study involving runway running trials and mechanical shoe testing	Innovative Running Shoes (IRS) featuring a 16-mm heel-to-toe drop and a three-layer midsole structure (upper and lower layers for cushioning, middle layer for support)	3D kinematics (joint angles/velocities), kinetics (ground reaction forces, loading rates, joint moments), mechanical properties (impact acceleration, longitudinal bending stiffness), and subjective perception (visual analogue scale)	IRS significantly reduced average and peak vertical loading rates, peak vertical force, and peak braking force compared to normal running shoes. The IRS also induced higher ankle dorsiflexion at touchdown and reduced knee internal rotation. Participants subjectively perceived the IRS to have superior cushioning
9	Song et al., (2024) [12]	China and Hungary	Single male participant (age 28, 70 kg) for CT scans and gait validation	3D foot-shoe coupled finite element (FE) model simulation	Carbon-fibre plates (CFP) with varying shapes (flat vs. curved) and stiffness levels (1-, 2-, and 3-mm thickness)	Peak forefoot plantar pressure, metatarsal bone stress (von Mises), and MTP joint contact force transmission	Increasing CFP stiffness reduced peak plantar pressure, with curved plates showing a further reduction of 5.51% to 12.62% compared to flat plates. While CFP designs had little effect on the high stress of the 2nd and 3rd metatarsals, they reduced stress in other metatarsals and promoted a more uniform force transmission pathway
10	Engel et al. (2024) [34]	Germany	10 male trained runners (average age 26.7 years)	Randomized controlled cross-over study	Downward-curved carbon fibre insoles compared to butyl rubber control insoles (both worn in Saucony Fastwitch 9 road racing shoes)	Oxygen uptake (running economy), 3-km time trial performance, time to exhaustion, heart rate, blood lactate, stride frequency, stride length, and perceived shoe comfort/performance	No significant benefits were found for running performance, physiology, or biomechanics. Shoe comfort was significantly impaired when using the carbon fiber insoles compared to the control condition.
11	Franz., et al. (2012) [16]	USA	12 healthy males with substantial barefoot running experience and midfoot strike preference	Randomized, single-session repeated measures design using treadmill running at 3.35 m/s	Barefoot vs. lightweight cushioned shoes (Nike Mayfly, ~ 150 g) with incremental added mass (lead strips up to 450 g)	Rates of oxygen consumption (*VO*˙_2_​), carbon dioxide production (*VCO*˙_2_​), gross metabolic power (W/kg), and stride length	Adding 100 g of mass per foot increases metabolic cost by ~ 1% regardless of footwear. Barefoot running offers no metabolic advantage over lightweight cushioned shoes; when mass is equalized, shod running is 3%-4% more economical than barefoot running due to shoe cushioning
12	Tung., et al. (2014) [17]	USA	12 healthy runners (10 males, 2 females) who preferred a midfoot strike pattern and had substantial barefoot/minimalist running experience	A repeated-measures experimental design. Subjects completed a standing trial and four 5-minute running trials at a speed of 3.35 m/s in a randomized order	The study evaluated unshod running on a normal rigid treadmill belt, unshod running on 10-mm and 20-mm thick midsole foams slats (Phylite) attached to the belt, and shod running in lightweight, cushioned shoes (Nike Free 3.0 V2, ~ 211 g)	Metabolic power demand (W/kg), gross oxygen consumption (*V*˙*O*2​), stride frequency, and ground contact time.	Running unshod on 10-mm foam reduced metabolic power demand by 1.63% compared to the rigid belt, while 20-mm foam showed no significant effect. Additionally, the metabolic cost of running shod and unshod on a rigid surface was similar. This is because the energetic benefits of cushioning counterbalanced the metabolic penalty of added shoe mass.
13	Kulmala et al. (2018) [18]	Finland	12 healthy men (mean age 27) with several years of sports experience and a heel strike running pattern	A randomised 3D running analysis using an eight-camera motion system and force platforms at two specific training speeds: 10 km/h and 14.5 km/h	Highly cushioned maximalist (MAX) shoes (Hoka Conquest) compared to conventional (CON) shoes (Brooks Ghost 6)	Vertical ground reaction force impact peak (IP), loading rate (LR), leg stiffness, and leg compression	Highly cushioned maximalist shoes increased leg stiffness and amplified impact loading compared to conventional shoes. At the faster speed (14.5 km/h), the impact peak and loading rate were 10.7% and 12.3% greater, respectively, in the maximalist shoe. The runners’ legs became stiffer during landing when wearing maximalist shoes, which countered the intended impact attenuation of the extra cushioning.
14	Madsen et al., (2025) [35]	Denmark	10 trained and highly trained male distance runners (half-marathon time: 75 ± 3 min)	Randomized, crossover, and counterbalanced design. Participants performed preliminary graded treadmill tests and three separate 80-min runs followed by post-exercise graded tests	Top-tier carbon-plated shoe with highly responsive foam (CP-shoe; Nike Alphafly 3) versus a non-carbon training shoe (NCP-shoe; Nike Pegasus 41)	Running economy (RE), running speed at lactate threshold (LT-speed), blood lactate concentration ([La−]b), heart rate (HR), and rate of perceived exertion (RPE}	The CP-shoe improved RE and lowered [La−]b, HR, and RPE compared to the NCP-shoe at matched external workloads. At matched internal workloads, the CP-shoe maintained superior RE and lower HR. Contrary to hypotheses, RE and LT-speed improved after 80 min of running in both conditions, with the CP-shoe consistently yielding higher LT-speeds (~ 0.6 km/h) than the NCP-shoe.

*VO*₂ Oxygen consumption, *VCO*₂ Carbon dioxide production, *VO*₂*max* Maximal oxygen uptake, *GRF* Ground reaction force, *MTP* Metatarsophalangeal joint, *EMG* Electromyography, *VALR* Vertical average loading rate, *CFP* Carbon-fibre plate, *LBS* Longitudinal bending stiffness, *MBS* Midsole bending stiffness, *pMTU* plantar muscle-tendon unit, *sMTU* shank muscle-tendon unit, *IRS* Innovative running shoes, *AFT* Advanced footwear technology, *CON* Conventional shoes, *MAX* Maximalist shoes, *NP* Nike prototype shoes, *NVF* Nike Vaporfly, *NZM* Nike Zoom Matumbo, *ADI* Adidas Adizero Adios, *FE* Finite element, *RE* Running economy, *LT-speed* Running speed at lactate threshold, *HR* Heart rate, *RPE* Rate of perceived exertion, *IP* Impact peak, *LR* Loading rate, *CoM* Center of mass

### Methodological quality of the included studies

The methodological quality of the included studies was assessed using an adapted risk-of-bias evaluation approach to account for the heterogeneity of study designs included in this review. While the Cochrane Risk of Bias framework is widely recommended for evaluating bias in randomized controlled trials [23, 24], the present review included a combination of randomized crossover trials, experimental laboratory studies, quasi-experimental designs, and biomechanical simulation models. Therefore, the assessment framework was adapted to ensure appropriate evaluation across diverse study types. The evaluation focused on key domains including study design quality, participant selection, measurement validity, reporting transparency, and potential sources of bias. Each study was assessed and categorized as having low risk of bias, some concerns, or high risk of bias based on these criteria. Experimental and randomized crossover studies were evaluated with greater emphasis on internal validity, whereas biomechanical and simulation studies were assessed in terms of model validity and methodological transparency. This adapted approach ensured that all included studies were assessed in a manner appropriate to their methodological design, thereby providing a more accurate and meaningful interpretation of the overall evidence base.

### Compliance and registration

The methodological procedures of this systematic review were conducted in accordance with internationally recognised guidelines for systematic reviews, particularly the PRISMA statement [20]. These guidelines were followed throughout the stages of literature search, study selection, data extraction, and reporting of results to ensure transparency and methodological rigour. Although the review followed PRISMA recommendations, the study protocol was not formally registered in an external registry, such as PROSPERO, because the review was conducted as part of an academic research synthesis on emerging footwear technologies in sports biomechanics.

### Summary measures

The primary summary measures considered in this review included indicators related to running economy, biomechanical performance, physiological responses, and running performance outcomes. Running economy was typically quantified through metabolic indicators such as oxygen uptake (VO₂), carbon dioxide production (VCO₂), or metabolic power expressed relative to body mass. In exercise physiology, running economy is commonly defined as the energy demand for running at a given submaximal velocity, typically measured through steady-state oxygen consumption during treadmill running [1, 2]. Lower oxygen consumption at a given speed reflects greater metabolic efficiency and is considered a key determinant of endurance running performance. Biomechanical measures included ground reaction forces, loading rates, joint kinematics and kinetics, plantar pressure distribution, and muscle activation patterns. These biomechanical variables are widely used in running research to examine how runners interact mechanically with the ground and how footwear or technique modifications influence movement efficiency and injury risk [25, 26]. Ground reaction forces, in particular, provide important information about force transmission and joint loading during running. Physiological variables examined across studies included heart rate, blood lactate concentration, respiratory exchange ratio, and rate of perceived exertion. These measures are commonly used to assess metabolic and cardiorespiratory responses during endurance exercise and help explain how biomechanical or equipment modifications influence physiological efficiency [27]. Performance outcomes were evaluated using indicators such as running velocity, time-trial performance, time to exhaustion, or predicted improvements in endurance running performance. Such metrics are widely employed in sports science research to determine the practical performance benefits of biomechanical or technological interventions in running.

### Synthesis of results

Due to substantial heterogeneity across the included studies, a quantitative meta-analysis was not performed. The heterogeneity was evident in multiple domains, including study design (randomized crossover trials, laboratory experiments, and biomechanical simulations), participant characteristics (recreational, trained, and elite runners), footwear technologies (carbon-fibre plates, cushioning systems, stiffness modifications, and minimalist designs), and outcome measures (biomechanical, physiological, and performance variables). Given these variations, pooling data statistically would not provide meaningful or valid summary estimates. Therefore, a qualitative synthesis approach was adopted to systematically interpret patterns and relationships across studies.

### Publication bias

Publication bias was considered a potential limitation of the present review. In many areas of scientific research, including sports science, studies reporting statistically significant or positive findings are more likely to be published than those reporting null or negative results, which may influence the overall interpretation of evidence [28]. This phenomenon can lead to an overestimation of intervention effects when synthesizing published literature. Although the present review attempted to reduce this bias by including studies reporting neutral or non-significant findings, the possibility of publication bias cannot be completely excluded. The assessment of publication bias is typically conducted in systematic reviews using statistical approaches such as funnel plot analysis or regression-based methods [22, 29]. However, because the present review did not conduct a meta-analysis and included a relatively small number of studies, formal statistical tests for detecting publication bias were not applied. Consequently, the findings of this review should be interpreted with appropriate caution, acknowledging the potential influence of selective publication within the available literature.

### Additional analyses

Additional analyses were conducted through subgroup interpretation of footwear technologies and their associated biomechanical mechanisms. The included studies were categorised according to the type of footwear innovation investigated, including carbon-fibre plates, midsole stiffness modifications, cushioning systems, and minimalist shoe designs. This categorisation allowed for a more detailed examination of how different technological components influence running biomechanics and performance. The synthesis also considered contextual factors such as running environment, terrain conditions, and participant characteristics to better interpret variations in study findings. These additional analyses helped identify patterns in the interaction between footwear design and running mechanics, contributing to a deeper understanding of how emerging footwear technologies influence endurance performance and injury risk.

## Results

In the present review, results are reported based on direct findings from the included studies. Where interpretations regarding potential performance implications or biomechanical mechanisms are presented, these are explicitly derived from the original authors’ interpretations or are clearly indicated as inferred outcomes based on reported data. Efforts were made to distinguish between directly measured outcomes and inferred or theoretical implications to maintain clarity and avoid overinterpretation. A total of fourteen studies met the predefined inclusion criteria and were included in the qualitative synthesis. The included studies investigated a range of emerging footwear technologies, including carbon-fibre plates, advanced midsole foams, midsole stiffness modifications, cushioning systems, and minimalist shoe designs. Across these studies, outcome measures primarily focused on running economy, biomechanical variables, physiological responses, and performance-related indicators. Most studies employed controlled laboratory experiments or randomized crossover designs involving recreational, trained, or elite runners. The synthesised findings revealed varying effects of different footwear technologies on running efficiency, biomechanical behaviour, and physiological responses, as summarized in Tables 2, 3, 4 and 5.

**Table 3:** Risk of Bias Assessment of Included Studies *n* = 14

Study	Randomization Process	Deviations from Intended Intervention	Missing Outcome Data	Measurement of Outcome	Selection of Reported Results	Overall Risk of Bias	Weight in Evidence Interpretation
Hoogkamer et al., (2018) [7]	Low Risk	Low Risk	Low Risk	Low Risk	Low Risk	Low Risk	Strong evidence supporting improved running economy with advanced footwear technology
Barnes & Kilding, (2019) [8]	Low Risk	Low Risk	Low Risk	Low Risk	Low Risk	Low Risk	High-quality experimental evidence supporting improved running economy
Healey & Hoogkamer, (2022) [14]	Some Concerns	Low Risk	Low Risk	Low Risk	Low Risk	Some Concerns	Evidence reliable but interpretation should consider small sample size
Corbí-Santamaría, P., et al. (2025) [30]	Low Risk	Low Risk	Low Risk	Low Risk	Some Concerns	Some Concerns	Results informative for mountain running biomechanics but limited generalizability
Yang et al. (2020) [31]	Low Risk	Some Concerns	Low Risk	Low Risk	Some Concerns	Some Concerns	Moderate evidence regarding gait retraining combined with minimalist footwear
Xu et al. (2025) [32]	Low Risk	Low Risk	Low Risk	Low Risk	Some Concerns	Some Concerns	Useful biomechanical insights into carbon plate geometry effects
Cigoja et al. (2020) [10]	Low Risk	Low Risk	Low Risk	Low Risk	Low Risk	Low Risk	High confidence in biomechanical findings related to midsole stiffness
Fu et al. (2022) [33]	Some Concerns	Low Risk	Low Risk	Low Risk	Low Risk	Some Concerns	Reliable biomechanical findings but limited by moderate sample size
Song et al., (2024) [12]	High Risk	Low Risk	Low Risk	Low Risk	Some Concerns	High Risk	Evidence interpreted cautiously due to single-participant simulation design
Engel et al. (2024) [34]	Low Risk	Low Risk	Low Risk	Low Risk	Low Risk	Low Risk	Strong experimental evidence for lack of performance benefit with carbon insoles
Franz., et al. (2012) [16]	Some Concerns	Low Risk	Low Risk	Low Risk	Low Risk	Some Concerns	Reliable metabolic findings but small sample size limits external validity
Tung., et al. (2014) [17]	Some Concerns	Low Risk	Low Risk	Low Risk	Low Risk	Some Concerns	Useful insights into cushioning and metabolic cost
Kulmala et al. (2018) [18]	Low Risk	Low Risk	Low Risk	Low Risk	Low Risk	Low Risk	High confidence evidence regarding maximalist cushioning biomechanics
Madsen et al., (2025) [35]	Low Risk	Low Risk	Low Risk	Low Risk	Low Risk	Low Risk	Strong physiological evidence supporting carbon-plated shoes

**Table 4:** Outcome measures and direction of effects across included studies

Study	Footwear Technology	Running Economy / Metabolic Cost	Biomechanical Variables	Physiological Variables		Performance Outcomes	Overall Effect Direction
Hoogkamer et al., (2018) [7]	Nike prototype (NP; later Nike Vaporfly): carbon-fibre plate + PEBA (ZoomX) foam vs. Nike Zoom Streak 6 (EVA + air bag) and Adidas Adizero Adios BOOST 2 (TPU foam); NP → ↑ compliance and ↑ energy return (~ 87%)	NP → ↓ metabolic cost ( ~ − 4%) vs. NS and AB across speeds (14–18 km·h⁻¹)	↑ peak vertical GRF (+ 1.1%); ↓ step frequency ( ~ − 0.6–0.8% → longer steps); ↑ contact time ( ~ + 0.6%)	Measured: VO₂, blood lactate (< 4 mmol·L⁻¹), RER (< 0.9)		Predicted ↑ running velocity ( ~ + 3.4%) at marathon pace due to ↓ energetic cost	Positive / Performance-enhancing - carbon plate + PEBA foam ↓ metabolic cost (~ 4%) → improved endurance running performance potential.
Barnes & Kilding, (2019) [8]	Nike Vaporfly (NVF) vs. track spikes (Nike Zoom Matumbo 3, NZM) vs. marathon shoes (Adidas Adizero Adios 3, ADI); weight-matched condition (NVF+)	NVF → ↑ RE vs. NZM (+ 2.6 ± 1.3%); NVF → ↑ RE vs. ADI (+ 4.2 ± 1.2%); NVF+ → ↑ RE vs. ADI (+ 2.9 ± 1.3%)	Measured across running velocities; correlations between biomechanical changes and RE improvements → small / unclear (↔)	Measured: VO₂, VCO₂, VO₂max		↑ racing efficiency; NVF considered suitable for both road and track racing	Positive - NVF technology ↑ running economy compared with traditional track spikes and marathon racing shoes.
Healey & Hoogkamer, (2022) [14]	Nike Vaporfly 4% intact (VFintact) vs. reduced stiffness condition (VFcut; carbon plate with six cuts → ↓ LBS ~ 66% flexion, ~ 72% extension)	↔ running economy; VFcut showed only + 0.55% higher metabolic rate vs. VFintact (not significant)	VFcut → ↑ MTP dorsiflexion; ↑ MTP angular velocity; ↑ MTP negative work; VFintact → ↑ contact time ( ~ + 1%); ↔ step frequency and peak vertical GRF	↔ VO₂ and VCO₂-derived metabolic rate (no significant differences)		↔ expected performance benefits maintained despite reduced LBS	Neutral - reducing carbon-plate LBS alone did not significantly affect running economy or overall biomechanics, suggesting energy savings arise from combined shoe design features (foam + geometry + plate).
Corbí-Santamaría, P., et al. (2025) [30]	Advanced Footwear Technology (AFT): curved carbon-fibre plate + reactive midsole foams vs. Conventional (CON) mountain running shoes	↔ running economy (no improvement with AFT in mountain running conditions)	↓ step frequency; ↑ vertical oscillation of CoM; slight ↑ contact time; slight ↑ step length.	↔ heart rate; ↔ blood lactate concentration (no significant differences vs. CON).		↔ time-trial performance (5.19 km mountain circuit); no improvement in total or segment times.	Neutral / Context-dependent - AFT altered running biomechanics (↓ cadence, ↑ vertical oscillation) but did not improve performance or physiological responses in mountain running conditions.
Yang et al. (2020) [31]	Minimalist shoes (INOV-8 Bare-XF 210 V2): 3 mm outsole, 0 mm heel-toe drop, no midsole, 227 g; groups: GR (gait retraining + minimalist shoes) vs. MIN (minimalist shoes only)	Not directly measured; ↑ vertical stiffness (GR + 17.2%, MIN + 7.1%) → potential ↑ running economy	↓ loading rate (both groups; greater in GR); GR → forefoot strike adoption (78%), ↓ foot-strike angle; ↑ ankle plantarflexion; ↑ hip extension velocity; ↑ peak ankle moment (GR); ↓ knee extension moment; ↓ hip power	N/A (traditional physiological markers such as HR or VO₂ not measured)		Potential ↑ running efficiency and ↓ injury risk due to ↓ impact forces and improved vertical stiffness	Positive (especially GR condition) - gait retraining + minimalist footwear ↓ impact loading and ↑ vertical stiffness, suggesting improved running mechanics and potential performance benefits.
Xu et al. (2025) [32]	Carbon-fibre plate shoes (CPS): Flat plate (ASICS METASPEED SKY PARIS) vs. Curved plate (ASICS METASPEED EDGE PARIS)	Not directly measured; literature suggests CCFP ↑ RE (~ 3.45%) vs. FCFP (~ 0.19%), but current study → ↔ joint power/work	Curved plate → ↓ hip contact angle; ↓ knee contact angle; ↓ hip flexion moment; Muscle activation differences in TA, MG, LG (fatigue interaction during late stance)	Measured: HR; RPE; EMG (TA, MG, LG) to confirm running-induced fatigue		Curved plate → potential ↑ running performance and ↓ joint loading → possible ↓ injury risk	Positive - curved CFP ↓ hip/knee joint loads and optimizes gait mechanics, especially under fatigue conditions.
Cigoja et al. (2020) [10]	↑ Midsole Bending Stiffness (MBS) via carbon fiber plate insertion (1.2 N·mm⁻¹ → 11.9 N·mm⁻¹)	Not directly measured; potential ↑ efficiency inferred from ↓ MTU shortening velocity and altered force-generation mechanics	↓ arch deformation; ↓ arch flexion velocity; ↓ pMTU shortening & velocity; ↓ sMTU shortening velocity; ↓ positive & net work at arch/pMTU; ↑ contact time ( ~ + 13 ms)	Hypothesized ↓ muscle force generation rate (~ 4.76%) → potential ↓ motor unit recruitment		Potential ↑ locomotion efficiency → is associated with endurance running performance	**Positive** - ↑ MBS → ↓ MTU mechanical work and improved locomotion efficiency.
Fu et al. (2022) [33]	Innovative Running Shoes (IRS): ↑ heel-to-toe drop (16 mm), three-layer midsole (cushioning-support-cushioning), ↑ longitudinal bending stiffness, thicker rearfoot (32 mm) vs. Normal Running Shoes (NRS; HTD 6.5 mm, rearfoot 18 mm)	Potential ↑ efficiency (~ 1–2%) associated with ↑ LBS and ↑ HTD (mechanical efficiency inference)	Kinematics: ↑ ankle dorsiflexion at touchdown; ↓ knee internal rotation; ↓ MTP joint ROM and dorsiflexion velocity Kinetics: ↓ average & peak vertical loading rate; ↓ peak vertical force; ↓ peak braking force; ↓ GRF during stance	N/A (physiological markers not directly measured)		↑ cushioning perception; ↑ propulsion efficiency; ↓ knee internal rotation → potential ↓ injury risk	Positive - IRS ↑ cushioning & motion control → ↓ impact loading and improved lower-limb biomechanics in female runners.
Song et al., (2024) [12]	Carbon-fibre plate (CFP) shoes: Flat plate (FCFP) vs. Curved plate (CCFP); ↑ longitudinal bending stiffness (varying plate thickness)	CCFP → ↑ RE ( ~ + 3.45%); FCFP → minimal RE improvement ( ~ + 0.19%)	↓ peak forefoot plantar pressure; ↓ metatarsal stress (1st, 4th, 5th); improved load distribution; ↓ MTP joint energy loss	N/A (biomechanical simulation; physiological responses not directly measured)		Potential ↑ distance running performance; ↓ forefoot loading → possible ↓ overuse injury risk	**Positive** - ↑ CFP stiffness (especially curved plates) → ↓ plantar pressure (≈ 5.5–12.6%) and improved load distribution during running
Engel et al. (2024) [34]	Downward-curved carbon fiber insoles (↑ longitudinal bending stiffness: ~9 → ~38 N·mm⁻¹) inserted in racing shoes (Saucony Fastwitch 9) vs. mass-matched butyl rubber control insoles	↔ VO₂ at submaximal speeds (10–16 km·h⁻¹); carbon insoles showed non-significant ↑ VO₂ (+ 2.9–5.7%)	↔ stride frequency; ↔ stride length (no significant differences)	↔ HR; ↔ RER; ↔ blood lactate (no significant differences)		↔ time-to-exhaustion; ↔ 3-km time trial performance; ↓ perceived comfort with carbon insoles	Neutral ↑ bending stiffness via carbon insoles did **not** improve economy, biomechanics, physiology, or performance; ↓ comfort was reported.
Franz., et al. (2012) [16]	Barefoot vs. lightweight cushioned shoes (Nike Mayfly, ~ 150 g); added mass conditions (+ 150 g, + 300 g, + 450 g) to control for footwear weight	↑ metabolic cost ≈ + 1% per + 100 g added mass (both conditions); When mass equalized → shod running ↑ economy (+ 3.3–4.2%) vs. barefoot	Participants: habitual midfoot strikers; Shod running → ↑ stride length (+ 3.3%)	Measured: VO₂, VCO₂, RER, gross metabolic power (W·kg⁻¹)		Barefoot → no metabolic advantage; lightweight cushioned shoes (~ 129 g predicted optimal) may improve running efficiency	Positive for shod running - cushioning ↓ metabolic demand when shoe mass is controlled (cost-of-cushioning principle)
Tung., et al. (2014) [17]	Unshod rigid surface vs. unshod + foam cushioning (10 mm, 20 mm Phylite) vs. lightweight cushioned shoes (Nike Free 3.0 V2, ~ 211 g)	10 mm cushioning → ↓ metabolic power (− 1.63%) & ↓ VO₂ (− 1.47%) vs. rigid surface; 20 mm cushioning → ↔ metabolic cost; Shod vs. unshod (rigid) → ↔ metabolic demand	Shod running → ↓ stride frequency (− 2.5%), ↑ ground contact time (+ 5.9%); Unshod conditions (0, 10, 20 mm) → ↔ stride parameters	Measured: VO₂, VCO₂, RER, metabolic power (W·kg⁻¹)		Moderate cushioning → ↓ metabolic demand during submaximal running; excessive cushioning may negate benefits	**Mixed**: Moderate cushioning (10 mm) ↑ running economy; excessive cushioning (20 mm) ↔ effect; shoe mass may offset cushioning benefits
Kulmala et al. (2018) [18]	MAX (Hoka Conquest): 43 mm heel midsole ↑ cushioning vs. CON (Brooks Ghost 6): 33 mm heel midsole	RE / Metabolic Cost: Not assessed (N/A); Study highlights importance of elastic leg behaviour for efficient force production	Leg stiffness: ↑ in MAX shoes (especially at 14.5 km/h) Impact loading: ↑ IP (+ 10.7%) and ↑ LR (+ 12.3%) in MAX vs. CON Leg compression: ↓ (~ 3.1–3.2 mm) in MAX CoM movement: ↑ CoM height; ↓ maximal CoM descent during stance Step parameters: ↔ cadence, step length, contact time (no significant difference)	N/A (not assessed); study involved 12 healthy male runners (mean age 27) with a consistent heel-strike pattern	MAX shoes ↑ impact loading → potential ↑ risk of impact-related injuries at higher speeds		Negative / Counterintuitive - MAX cushioning → ↑ leg stiffness → ↑ impact loading (IP, LR), especially at higher speeds
Madsen et al., (2025) [35]	CP-shoe (Nike Alphafly 3; carbon plate + high energy-return foam) vs. NCP-shoe (Nike Pegasus 41; conventional trainer)	↑ RE (+ 2–6%; ~4.5% well-rested) in CP-shoe vs. NCP; RE improved during 80-min run in both conditions	Attributed to ↑ longitudinal bending stiffness (carbon plate) + ↑ energy return from compliant midsole foams	↓ VO₂; ↓ blood lactate (− 0.3–0.5 mmol·L⁻¹); ↓ HR (− 4–5%); ↑ LT speed (+ 0.5–0.6 km·h⁻¹)		↑ endurance performance potential; ↓ perceived exertion (Borg scale)	Positive / Enhancing - CP-shoe ↑ RE, ↑ LT speed, ↓ HR & lactate → improved endurance running efficiency during prolonged running

↑ increase; ↓ decrease; ↔ no significant change; ~ approximately; ± standard deviation

*RE* Running economy, *VO*₂ Oxygen consumption, *VCO*₂ Carbon dioxide production, *HR* Heart rate, *RPE* Rate of perceived exertion, *LT-speed* Running speed at lactate threshold, *GRF* Ground reaction force, *MTP* Metatarsophalangeal joint, *EMG* Electromyography, *VALR* Vertical average loading rate, *CoM* Center of mass, *CFP* Carbon-fibre plate, *LBS* Longitudinal bending stiffness, *MBS* Midsole bending stiffness

**Table 5:** Summary of footwear technologies and their biomechanical effects

Footwear Technology	Structural Characteristics	Biomechanical Mechanisms		Observed Biomechanical Effects	Implications for Performance	Potential Injury Implications	Representative Studies
Carbon-fibre plate shoes (super shoes)	Full-length carbon plate embedded in highly compliant midsole foams (e.g., PEBA)	Increased longitudinal bending stiffness and improved energy return during push-off	↓ MTP joint energy loss, ↓ joint work at ankle and foot, slight ↓ step frequency with longer stride length		↑ running economy (≈ 2–6%) and improved endurance running efficiency	Redistribution of forefoot loads may reduce localized stress but may alter joint loading patterns	Hoogkamer et al., 2018 [7]; Barnes & Kilding, 2019 [8]; Xu et al., 2025 [11]; Song et al., 2024 [12]; Madsen et al., 2025 [35]
Curved carbon-fibre plate geometry	Curved plate design positioned under forefoot with increased stiffness gradient	Alters forefoot bending angle and load transfer across the metatarsophalangeal joint	↓ hip and knee joint angles and moments; ↓ plantar pressure distribution across forefoot		is associated with running mechanics under fatigue conditions	Reduced forefoot pressure may decrease risk of metatarsal overuse injuries	Xu et al., 2025 [11]; Song et al., 2024 [12]
Midsole bending stiffness modification	Increased stiffness through embedded plates or structural reinforcement	Reduces deformation of foot arch and modifies muscle-tendon unit behavior	↓ arch deformation; ↓ muscle-tendon shortening velocity; ↑ ground contact time		Improved mechanical efficiency and reduced muscular work during stance phase	Potential redistribution of mechanical load within lower limb structures	Cigoja et al., 2020 [10]
Advanced midsole foams technology	Highly resilient, compliant foams with high energy return (e.g., PEBA / ZoomX)	Increased energy storage and return during ground contact	↓ metabolic cost of running; altered stride mechanics and contact times		Improved running economy and endurance performance	Reduced muscular demand may lower fatigue-related injury risk	Hoogkamer et al., 2018 [7]; Madsen et al., 2025 [35]
Minimalist footwear	Thin outsole, minimal cushioning, zero heel-to-toe drop, flexible structure	Encourages forefoot strike and modifies loading patterns	↓ loading rate; ↑ ankle plantarflexion; ↑ vertical stiffness		may indicate in running efficiency after adaptation	Reduced impact peaks may decrease certain overuse injuries but requires adaptation period	Yang et al., 2020 [15]
Innovative multi-layer midsole structures	Multi-layer cushioning and support systems with increased heel-toe drop	Improves shock absorption and stabilizes lower limb motion	↓ vertical loading rate; ↓ braking force; ↓ knee internal rotation		Improved propulsion and perceived cushioning	Reduced knee joint stress may lower risk of patellofemoral pain	Fu et al., 2022 [33]
Maximalist cushioning shoes	Extremely thick midsole cushioning (high stack height)	Increased cushioning alters neuromuscular control during landing	↑ leg stiffness; ↑ vertical impact loading despite increased cushioning		Limited or no metabolic advantage	Potential ↑ impact-related injury risk due to amplified loading	Kulmala et al., 2018 [18]
Carbon-fibre insoles (removable)	Stiff curved carbon plate inserted into conventional shoe	Increases bending stiffness without altering midsole foams properties	Minimal biomechanical changes; no improvement in stride mechanics		No significant improvement in running economy or performance	Reduced comfort reported by runners	Engel et al., 2024 [34]
Surface or shoe cushioning variations	Moderate cushioning vs. rigid surfaces or barefoot conditions	External cushioning reduces muscular effort needed to absorb impact	↓ metabolic cost with moderate cushioning; stride modifications		Moderate cushioning may improve running economy	Excessive cushioning or shoe mass may offset metabolic benefits	Tung et al., 2014 [17]; Franz et al., 2012 [16]
Advanced footwear technology in trail running	Carbon-plate shoes combined with reactive foam used in off-road terrain	Altered stride mechanics due to terrain interaction	↓ step frequency; ↑ vertical oscillation of center of mass		No clear performance benefit in mountain running conditions	Biomechanical changes may affect stability on uneven terrain	Corbí-Santamaría et al., 2025 [19]

Table 2 summarizes the characteristics of the fourteen studies included in this systematic review, highlighting differences in participants, study designs, footwear technologies, and outcome measures. Most studies used controlled laboratory experiments or randomized crossover designs involving recreational, trained, or elite runners. The investigations primarily focused on emerging footwear technologies such as carbon-fibre plates, advanced midsole foams, midsole stiffness modifications, minimalist footwear, and maximalist cushioning systems.

Key outcomes included running economy, biomechanical variables (e.g., ground reaction forces and joint mechanics), physiological responses (e.g., heart rate and blood lactate), and performance indicators. Several studies reported improvements in running economy and efficiency with carbon-plated footwear, whereas other designs produced mixed or context-dependent effects. Overall, the findings indicate that footwear technology influences running performance through complex interactions between shoe structure, biomechanics, and physiological responses.

Table 3. The risk of bias assessment indicated that the overall methodological quality of the included studies was generally acceptable, with most studies demonstrating low risk of bias across the evaluated domains. Several studies were classified as low risk because they employed well-controlled experimental or randomized crossover designs, clearly reported outcome measurements, and showed minimal concerns related to missing data or deviations from intended interventions. The findings derived from these studies were therefore considered to provide the most reliable evidence regarding the effects of sports footwear technologies on running performance and biomechanics. Several studies were rated as having some concerns, primarily due to limitations such as relatively small sample sizes, incomplete reporting of randomization procedures, or minor uncertainties in the selection of reported outcomes. The overall methodological quality of the included studies was generally acceptable. Most studies demonstrated low risk of bias, particularly those employing controlled experimental or randomized crossover designs with clearly reported outcome measures. Several studies presented some concerns, primarily due to small sample sizes or minor reporting limitations. One study was classified as high risk of bias due to methodological constraints associated with simulation-based design. Overall, greater interpretative weight was given to studies with lower risk of bias during evidence synthesis.

Although these methodological limitations do not invalidate the findings, they suggest that the evidence from these studies should be interpreted with moderate caution and considered supportive rather than definitive. One study was categorised as having a high risk of bias because of substantial methodological limitations related to study design and participant representation, which restrict the generalizability of its findings. In synthesizing the evidence, greater weight was therefore given to studies with lower risk of bias, while findings from studies with some concerns or higher risk were interpreted more cautiously and used primarily to complement the stronger evidence base. This approach ensured that the conclusions of the review were informed by the methodological rigour of the included studies and helped minimise the influence of potential bias on the overall interpretation of the results.

Table 4. The synthesis of outcome measures across the included studies indicates that advanced footwear technologies generally exert a positive influence on running economy and biomechanical efficiency, although the magnitude and consistency of these effects vary depending on the specific design characteristics of the footwear. Several studies demonstrated that carbon-fibre plate shoes combined with highly resilient midsole foams significantly improved running economy, often through reductions in metabolic cost and improvements in physiological indicators such as lower oxygen consumption, heart rate, and blood lactate levels. In addition to metabolic benefits, many footwear innovations including increased midsole stiffness, optimised plate curvature, and specialised midsole structures produced favourable biomechanical adaptations, such as reduced joint loading, decreased impact forces, improved load distribution, and more efficient muscle-tendon mechanics.

Statements referring to “potential” or “inferred” performance benefits are based on biomechanical or physiological indicators reported in the original studies and do not necessarily represent directly measured performance outcomes. Some interventions, such as isolated increases in plate stiffness or the use of carbon-fibre insoles without advanced foam technology, showed neutral effects on running economy and performance, suggesting that performance improvements arise from the combined interaction of multiple shoe design elements rather than a single component. Furthermore, certain footwear designs, particularly highly cushioned maximalist shoes in some conditions, produced counterintuitive biomechanical responses, including increased leg stiffness and higher impact loading, which may elevate injury risk at higher running speeds. Overall, the evidence suggests that while modern footwear technologies can enhance endurance running efficiency and alter biomechanical patterns, their effectiveness is context-dependent and influenced by the specific combination of cushioning, stiffness, geometry, and running environment.

In Table 5, the synthesis of footwear technologies across the included studies indicates that modern running shoe innovations influence biomechanical behaviour through multiple interacting mechanisms rather than a single structural feature. Carbon-fibre plates combined with highly resilient midsole foams consistently demonstrated the most pronounced effects, improving running economy and modifying joint mechanics by enhancing energy return and reducing muscular work during the stance phase.

Variations in plate geometry and midsole stiffness further influenced load distribution across the lower extremities, often reducing plantar pressure and joint moments. In contrast, minimalist footwear primarily altered running mechanics by encouraging forefoot strike patterns and increasing vertical stiffness, while highly cushioned maximalist shoes occasionally produced counterintuitive biomechanical responses such as increased leg stiffness and impact loading. Additional footwear innovations, including multi-layer midsoles and removable carbon insoles, showed mixed or context-dependent effects on running mechanics and performance. Overall, the evidence suggests that the interaction between shoe stiffness, cushioning properties, geometry, and running environment plays a critical role in determining the biomechanical outcomes associated with sports footwear technologies.

## Discussion

It is important to note that some interpretations presented in this review are based on inferred relationships between biomechanical variables and performance outcomes, rather than direct performance measurements, and should therefore be interpreted with appropriate caution. The present systematic review synthesised evidence from fourteen experimental and biomechanical studies examining the effects of emerging footwear technologies on running performance, biomechanics, and physiological responses. While the findings indicate that modern running shoe innovations, including carbon-fibre plate designs, advanced midsole foams materials, altered plate geometry, and innovative midsole structures substantially influence running mechanics, the evidence does not support a universally consistent performance benefit. Rather, the magnitude and direction of these effects appear highly dependent on the interaction between shoe design, running context, and individual biomechanics.

One of the most prominent developments in modern sports footwear is the integration of carbon-fibre plates within highly resilient midsole foams. A consistent pattern across studies suggests improvements in running economy and endurance performance with this configuration. For instance, shoes combining a curved carbon-fibre plate with PEBA-based foam reduced metabolic cost by approximately 4% [7], while Barnes and Kilding (2019) reported improvements ranging from 2.6 to 4.2% compared with traditional footwear [8]. However, these findings should not be interpreted as universally applicable. In contrast to these positive outcomes, other investigations suggest that the magnitude of improvement varies considerably across individuals and testing conditions, indicating that the benefits are not solely attributable to the presence of a carbon plate. This inconsistency may be explained by the interaction between foam properties, shoe geometry, and runner-specific biomechanics rather than a single technological component [5, 6]. Within this framework, the observed improvements in running economy can be understood as the result of synergistic interactions between compliant midsole foams (input) and altered joint mechanics (mediator), rather than the independent effect of the carbon plate itself.

The physiological mechanisms underlying these improvements appear to involve reductions in muscular work and enhanced energy transfer across lower-limb joints. Madsen et al. (2025) reported that carbon-plated racing shoes reduced oxygen uptake, heart rate, and blood lactate while increasing lactate-threshold speed, supporting the notion that footwear can influence both biomechanical and metabolic efficiency [35]. However, it remains unclear whether these physiological benefits are sustained across prolonged use or under varying environmental conditions, limiting their generalizability beyond controlled settings [7]. While running economy is strongly associated with enhanced performance [2], recent evidence suggests that its translation into real-world outcomes is mediated by environmental, biomechanical, and individual variability [1, 36]. Therefore, the extent to which laboratory-based physiological gains consistently translate into competitive performance remains only partially resolved. This supports the proposed model, where biomechanical mediators such as reduced joint work and improved muscle-tendon efficiency translate into downstream physiological benefits, including reduced metabolic cost and improved endurance performance.

Despite strong support for carbon-plate footwear, the present review highlights that plate stiffness alone does not explain performance improvements. Experimental manipulation of longitudinal bending stiffness showed no significant change in running economy when stiffness was reduced [14]. This finding contrasts with earlier assumptions that stiffness is a primary driver of performance enhancement and suggests that energy savings emerge from a more complex interaction between plate geometry, foam compliance, and overall shoe structure. Therefore, the evidence challenges reductionist interpretations and supports the concept of an “integrated footwear system,” in which multiple design elements interact to influence performance [3]. From a conceptual perspective, this finding reinforces the model’s central premise that modifying a single structural component in isolation is insufficient to alter performance outcomes without concurrent changes in biomechanical mediators.

Beyond stiffness, plate geometry appears to play a critical role in shaping biomechanical outcomes. Curved carbon-fibre plates have been shown to reduce hip and knee joint moments and improve load distribution across the forefoot [11, 12]. However, these biomechanical advantages are not consistently associated with measurable performance gains. This discrepancy suggests that favourable mechanical changes do not always translate directly into improved running economy or endurance performance. One possible explanation is that biomechanical efficiency and metabolic efficiency are not always aligned, particularly under fatigue conditions. Indeed, fatigue-induced alterations in biomechanics, such as increased ground contact time and reduced ankle stiffness [37], may offset the mechanical benefits of optimized plate geometry. Within the proposed framework, plate geometry can be interpreted as a structural input that selectively modifies biomechanical mediators such as joint loading and force distribution; however, the translation of these changes into performance outcomes appears to depend on additional contextual and individual factors.

Midsole stiffness further complicates this relationship. Increasing midsole bending stiffness has been shown to reduce muscle-tendon unit shortening velocity and mechanical work [10], theoretically improving efficiency. However, in contrast to these findings, studies manipulating stiffness in isolation have reported minimal or no improvements in running economy [14]. This inconsistency suggests that stiffness-related benefits are conditional and may depend on how stiffness interacts with other shoe characteristics, such as foam compliance and runner-specific mechanics, rather than acting as an independent performance factor [9]. This inconsistency further supports the model by indicating that biomechanical changes alone do not guarantee performance improvements unless they effectively influence metabolic efficiency.

In contrast to carbon-plate technologies, other footwear interventions demonstrate more variable and context-dependent outcomes. Minimalist footwear, for example, consistently alters running biomechanics by promoting forefoot strike patterns and reducing impact loading [15]. However, these biomechanical changes do not consistently improve running economy. This divergence indicates that biomechanical alterations alone are insufficient to enhance performance and may require prolonged neuromuscular adaptation to become metabolically advantageous [38]. Within this framework, minimalist footwear primarily alters biomechanical mediators without consistently affecting performance outputs, highlighting a partial pathway within the model rather than a complete performance-enhancing mechanism.

Similarly, the role of cushioning in running economy reflects a complex trade-off between impact attenuation and energetic cost. Moderate cushioning has been shown to reduce metabolic demand [17], whereas excessive cushioning or increased shoe mass can negate these benefits by increasing the energy cost of leg swing [16]. This non-linear relationship highlights that more cushioning is not necessarily better and that optimal performance likely occurs within a specific range of cushioning and mass. This trade-off can be directly interpreted through the conceptual model, where competing effects at the structural level (cushioning vs. mass) produce opposing influences on biomechanical and physiological mediators.

Interestingly, highly cushioned maximalist footwear may produce counterintuitive biomechanical effects, as Kulmala et al. (2018) reported that increased midsole compliance can alter neuromuscular control and stiffness regulation rather than simply attenuating impact [18, 39, 40]. This paradox is likely explained by adaptive responses, whereby runners adjust leg stiffness and gait mechanics in response to altered surface compliance and shoe properties [41, 42]. Consequently, footwear effects should be interpreted within a neuromechanical framework, as human adaptation may offset or even reverse expected mechanical benefits [11].

Further evidence against isolated design interventions is provided by studies on carbon-fibre insoles. Although these devices increase shoe stiffness, they do not improve running economy, biomechanics, or performance and may reduce comfort [34]. In contrast to integrated carbon-plate footwear, these findings reinforce the idea that performance benefits arise from the synergy of multiple design elements rather than single-component modifications. Within the proposed framework, this further illustrates that increasing stiffness without optimizing other structural and biomechanical components fails to produce meaningful performance outcomes. Finally, the effectiveness of advanced footwear technologies is strongly influenced by environmental context, with benefits observed in road running often failing to translate to complex terrains. Recent evidence indicates that although carbon-plated footwear improves running economy under controlled conditions, its performance advantages are inconsistent in trail and mountain environments due to terrain-induced biomechanical variability [19, 43]. Notably, no significant performance improvements were observed despite biomechanical alterations in mountain running, suggesting that uneven terrain disrupts energy-return mechanisms and reinforces the context-dependent nature of footwear performance. This observation strongly reinforces the context-dependent nature of the conceptual model, where environmental factors act as moderators influencing the relationship between biomechanical mediators and performance outcomes.

Overall, the findings of the present review indicate that modern footwear technologies influence running biomechanics and physiology through complex and interacting mechanisms. While carbon-fibre plate shoes combined with resilient midsole foams provide the most consistent improvements in running economy, these benefits are neither universal nor solely attributable to a single design feature. Instead, the effectiveness of footwear technologies depends on the interaction between structural design, biomechanical adaptation, environmental conditions, and individual runner characteristics. Therefore, the proposed conceptual model provides a unifying framework to interpret both the positive and inconsistent findings in the literature, demonstrating that footwear performance is not universally determined by design innovation but by the dynamic interaction between structure, biomechanics, and context.

### Proposed conceptual interpretation of footwear performance

Building upon the synthesized evidence, the present review proposes that the performance benefits of modern running footwear should be understood within an integrated system framework, rather than as the result of isolated design features. Specifically, we demonstrate that performance enhancement emerges from the interaction between three key domains: (1) structural inputs (e.g., midsole foams resilience, carbon plate stiffness, shoe geometry), (2) biomechanical mediators (e.g., joint work redistribution, muscle-tendon behavior, stride mechanics), and (3) performance outputs (e.g., running economy, fatigue resistance, endurance performance).

Within this framework, carbon-fibre plate shoes do not universally improve performance; rather, their effectiveness is context-dependent, influenced by factors such as running speed, terrain, fatigue state, and individual biomechanics. This perspective explains why certain studies reported substantial improvements in running economy, while others observed neutral or negligible effects. Importantly, the evidence suggests that no single footwear component is sufficient to produce performance gains in isolation. Instead, optimal outcomes arise when shoe design elements are synergistically aligned to enhance energy return while minimizing biomechanical inefficiencies. Therefore, this review advances the position that modern “super shoes” should not be interpreted as universally performance-enhancing technologies, but rather as conditionally effective systems whose benefits depend on the interaction between athlete, environment, and design characteristics.

**Fig. 2:**
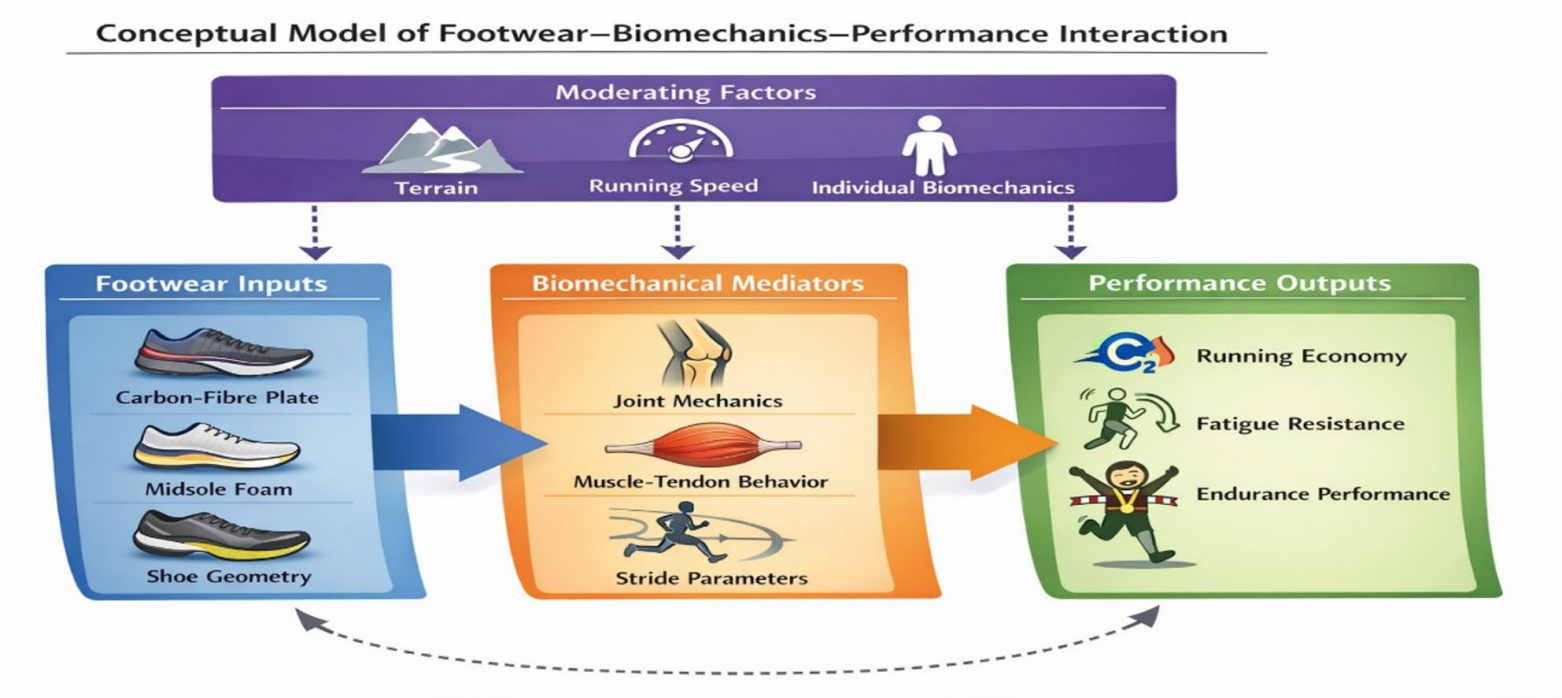
“Conceptual Model of Footwear-Biomechanics-Performance Interaction”

Figure 2 illustrates a conceptual model in which footwear inputs (carbon-fibre plate, midsole foams, geometry) influence performance indirectly through biomechanical mediators. These mediators, including joint mechanics, muscle-tendon behaviour, and stride parameters, determine how runners respond to footwear.

Performance outputs such as running economy, fatigue resistance, and endurance emerge as downstream effects of these adaptations. Importantly, moderating factors (terrain, running speed, individual biomechanics) influence all stages of this interaction. Thus, performance enhancement is context-dependent and arises from the integrated interaction of structure, biomechanics, and environment.

From a practical perspective, these findings suggest that footwear selection should be individualized based on the runner’s biomechanics, performance goals, and running environment. While advanced footwear technologies offer clear performance advantages in certain contexts, their biomechanical effects may vary substantially across different running conditions and athlete populations. Future research should therefore focus on long-term adaptation to footwear technologies, injury risk implications, and the interaction between footwear design and individual running mechanics.

Therefore, advanced running footwear (“super shoes”) should not be interpreted as inherently performance-enhancing devices, but rather as performance-modulating systems whose effects depend on the interaction between shoe design, biomechanics, and contextual factors.

### Critical appraisal of the evidence

Despite the overall positive findings, a critical evaluation of the included studies reveals several important methodological and contextual limitations that must be considered when interpreting the results. A key limitation across the literature is the restricted external validity of many studies. Most investigations were conducted under highly controlled laboratory conditions, typically using treadmill running protocols with relatively small sample sizes. While such designs enhance internal validity, they may not accurately reflect real-world running conditions, particularly in outdoor environments where terrain variability, environmental factors, and fatigue dynamics differ substantially. Consequently, the generalizability of these findings to competitive race settings remains limited. Furthermore, sample characteristics were often homogeneous, with a predominance of male, trained, or elite runners. This limits the applicability of findings to broader populations, including female athletes, recreational runners, and diverse anthropometric profiles. The lack of sex-specific analyses is particularly notable, given known biomechanical and physiological differences that may influence footwear responses. Another important concern relates to methodological variability across studies. Differences in running speeds, footwear models, testing durations, and outcome measures contribute to inconsistencies in findings. As a result, the evidence remains partially heterogeneous, making it difficult to establish definitive conclusions regarding the magnitude of performance benefits. Potential conflicts of interest and industry influence cannot be overlooked. Several studies evaluated commercially developed footwear technologies, particularly from major manufacturers. Although not always explicitly reported, the involvement of industry-funded research raises the possibility of bias toward positive findings, which may contribute to the overall favourable representation of advanced footwear technologies in the literature. Finally, the majority of studies focused on short-term biomechanical and physiological responses, with limited investigation of long-term adaptations or injury risk. Therefore, while acute improvements in running economy are well documented, the long-term implications of repeated use of such technologies remain insufficiently understood. Collectively, these limitations indicate that although the current evidence is promising, it should be interpreted with caution, and further high-quality, ecologically valid research is required.

### Practical implications for athletes, coaches, and footwear designers

The findings of this systematic review provide several practical insights for athletes, coaches, and footwear designers regarding the role of emerging footwear technologies in running performance and injury prevention. First, the consistent improvements in running economy observed in carbon-fibre plate shoes combined with highly resilient midsole foams suggest that such footwear may offer a meaningful competitive advantage for endurance runners. Studies included in the review demonstrated reductions in metabolic cost ranging from approximately 2% to 6%, which may translate into improved race performance, particularly in long-distance events where running economy is a critical determinant of success [7, 8, 35]. Consequently, elite and competitive distance runners may benefit from selecting footwear that incorporates advanced energy-returning materials and optimized plate geometries.

Second, coaches should consider the biomechanical adaptations associated with different footwear technologies when designing training programs. For example, footwear that increases midsole stiffness or incorporates carbon-fibre plates can alter lower-limb joint mechanics, stride parameters, and muscle activation patterns [10, 11]. These biomechanical changes may influence running technique and muscle loading patterns, which may require gradual adaptation to avoid potential overuse injuries. Similarly, minimalist footwear may encourage forefoot striking patterns and increased vertical stiffness, but the transition to such footwear should be carefully managed to allow sufficient musculoskeletal adaptation [15].

Third, footwear designers can use the biomechanical insights from the reviewed studies to optimize the interaction between shoe structure and human locomotion. Evidence suggests that performance benefits arise not from a single design feature but from the integration of multiple components, including midsole foams resilience, plate stiffness, shoe geometry, and cushioning characteristics. For instance, the combination of carbon-fibre plates with highly compliant foam materials appears to enhance energy storage and return during the stance phase, thereby reducing muscular work and improving running economy [6]. In contrast, excessively thick cushioning without appropriate structural design may lead to unintended biomechanical adaptations such as increased leg stiffness and impact loading [18]. These findings highlight the importance of optimizing the balance between cushioning, stiffness, and weight in future footwear designs.

Finally, athletes and practitioners should recognize that footwear performance effects are context-dependent. While advanced footwear technologies may provide substantial benefits during road running, their advantages may be reduced in trail or mountain running environments where surface irregularities and slope variations influence running mechanics [19]. Therefore, footwear selection should be tailored to the specific demands of the running environment, competition distance, and individual biomechanical characteristics. Given the strong involvement of commercial footwear manufacturers in the development and testing of advanced running shoes, the potential for industry-related bias toward positive findings cannot be excluded. This should be considered when interpreting the overall evidence base. Modern sports footwear technologies appear to influence running economy and biomechanics through complex and interacting mechanisms rather than isolated design features. While certain innovations, particularly carbon-fibre plate systems combined with advanced midsole foams, may enhance performance, their effects are not universal and depend on individual, biomechanical, and environmental factors. Therefore, footwear selection should be approached as an individualized and context-specific decision rather than a one-size-fits-all solution.

### Practical implications

From a practical perspective, the findings of this review suggest several important implications. Advanced footwear technologies, particularly those integrating carbon-fibre plates with compliant midsole foams, may improve running economy under controlled conditions. However, the extent of these performance benefits appears to depend on individual biomechanics, running context, and the interaction between multiple footwear components. Moreover, not all footwear innovations yield positive effects, as some designs demonstrate neutral or context-dependent outcomes. Therefore, athletes and coaches should adopt an individualized approach to footwear selection rather than assuming universal performance benefits across different populations and conditions.

### Research gaps and future directions

Despite significant advancements in footwear technology research, several important gaps remain in the current literature. First, there is a lack of long-term longitudinal studies examining how repeated use of advanced footwear influences musculoskeletal adaptation, injury risk, and performance over time. Most existing studies focus on acute responses, limiting understanding of chronic effects. Second, there is insufficient emphasis on individualized footwear responses. Future research should explore how factors such as foot morphology, running mechanics, and training status influence the effectiveness of specific footwear designs. Third, the literature lacks ecologically valid studies in diverse running environments, particularly in trail, mountain, and uneven terrain conditions. Current evidence is heavily biased toward treadmill and road running contexts. Fourth, there is a notable underrepresentation of female athletes and diverse populations, which limits the generalizability of findings across sexes and demographic groups. Finally, limited research has examined the interaction between footwear technology and injury risk, particularly under fatigue conditions and high training loads. Addressing these gaps will be essential for developing a more comprehensive understanding of how footwear technologies influence both performance and long-term athlete health.

### Limitations and future research directions

Several limitations of the present systematic review should be acknowledged. First, the relatively small number of included studies and their heterogeneity in design, participant characteristics, and outcome measures limited the possibility of conducting a quantitative meta-analysis. Second, although the review followed PRISMA guidelines and applied rigorous inclusion criteria, the reliance on published peer-reviewed studies may introduce publication bias, as studies reporting significant findings are more likely to be published. Third, the inclusion of only English-language articles may have excluded relevant studies published in other languages, potentially limiting the comprehensiveness of the evidence base. The synthesis was based primarily on short-term experimental studies, which restricts the ability to draw conclusions regarding long-term adaptations, injury risk, and real-world performance outcomes. Additionally, the relatively small number of included studies (*n* = 14) may limit the generalizability of the findings.

## Conclusion

This systematic review synthesized evidence from fourteen studies examining the biomechanical, physiological, and performance-related effects of emerging sports footwear technologies. Overall, the findings indicate that modern running shoe innovations, particularly carbon-fibre plates combined with highly resilient midsole foams, can significantly improve running economy and enhance endurance running performance by reducing metabolic cost and optimizing energy return during the stance phase of running. Several footwear technologies also demonstrated beneficial biomechanical adaptations, including reduced joint loading, improved load distribution, and more efficient muscle-tendon mechanics. However, the effects of footwear innovations were not uniform across all designs. Some interventions, such as isolated increases in plate stiffness or removable carbon-fibre insoles, showed limited or neutral effects on running performance, while highly cushioned maximalist footwear occasionally produced counterintuitive biomechanical responses that may increase impact loading. These findings suggest that the performance benefits of modern running shoes arise from the interaction of multiple structural elements, including midsole foams’ properties, plate geometry, stiffness, and cushioning, rather than a single technological feature. Moreover, the effectiveness of these technologies appears to be influenced by running conditions, terrain, and individual biomechanical characteristics. Therefore, while emerging footwear technologies offer promising advantages for endurance performance, their biomechanical effects remain context-dependent. Future research should focus on long-term adaptations, injury risk implications, and individualized footwear design to better understand how different shoe technologies can optimise running performance while minimizing injury risk.

## Data Availability

The datasets used and/or analysed during the current study are available from the corresponding author on reasonable request.

## Abbreviations

AFT: Advanced Footwear Technology
ADI: Adidas Adizero Adios
CFP: Carbon–Fibre Plate
CoM: Center of Mass
CON: Conventional Shoes
EMG: Electromyography
FE: Finite Element
GRF: Ground Reaction Force
HR: Heart Rate
IP: Impact Peak
IRS: Innovative Running Shoes
LBS: Longitudinal Bending Stiffness
LR: Loading Rate
LT: Speed–Running Speed at Lactate Threshold
MAX: Maximalist Shoes
MBS: Midsole Bending Stiffness
MIN: Minimalist Shoes
MTP: Metatarsophalangeal Joint
NP: Nike Prototype Shoes
NVF: Nike Vaporfly
NZM: Nike Zoom Matumbo
PEBA: Polyether Block Amide
RE: Running Economy
RER: Respiratory Exchange Ratio
RPE: Rate of Perceived Exertion
sMTU: Shank Muscle–Tendon Unit
pMTU: Plantar Muscle–Tendon Unit
TA: Tibialis Anterior
VALR: Vertical Average Loading Rate
VCO₂: Carbon Dioxide Production
VO₂: Oxygen Consumption
VO₂max: Maximal Oxygen Uptake
